# Many Interesting Paragraphs

**Published:** 1896-06

**Authors:** 


### Medical News and Miscellany.

---

**Dr. A. J. Gray** has removed from Wills Point to Ben Wheeler, his old home.

---

**Professor Seth M. Morris, M. D.,** of the University of Texas Medical College, with his wife and baby, are visiting Dr. Morris, Sr., at Austin.

---

**Errata.**—In Dr. F. S. White's paper in last issue, on page 614, for "Pierre," read "Pinel"; on page 616, eleventh line from top, for "creative," read "certain."

---

**Prof. J. M. Bodine, M. D.,** of the Medical Department of the University of Louisville, has been elected President of the American Medical College Association; a fitting compliment.

---

**The orchids** sent out by McDowell & Co., City of Mexico, see advertisement, are no trashy stuff, "twelve for a dollar," but are extra size, well rooted, flowering plants. We guarantee satisfaction.

---

**Married.**—In Waketon, Texas, on May 13th, 1896, Dr. D. F. Kirkpatrick to Miss Mattie Woodrum. The JOURNAL extends congratulations and best wishes to its old friend Kirkpatrick and his fair bride.

---

**The Third** International Congress of Dermatology will be held in London, August 4 to 8. Information may be had by addressing the secretary for the United States, Dr. Geo. Thomas Jackson, 14 E. 31st street, New York.

---

**Cap and Gown Again.**—The Kentucky School of Medicine will inaugurate, with this session, the wearing of the regular uniuersity cap and gown by faculty and graduating class. The

University of Pennsylvania and the Michigan University Medical schools use them.

---

**Dr. W. F. Blunt,** State Quarantine Officer at Galveston, had the misfortune last month to lose by fire his handsome residence just completed, at Lockhart, Texas. It cost $10,000. Insurance on house and contents only $4,800. The JOURNAL sympathises with the doctoɹ in his heavy loss.

---

**Dr. J. S. Steele,** of Dripping Springs, Texas, who is taking a thorough course of instruction in eye, ear, nose and throat at Chicago, has been tendered the position temporarily of first assistant surgeon in the Illinois Charity Eye and Ear Hospital Chicago. Steele has a career ahead of him; he'll get there.

---

**Dr. Charles A. L. Reed,** of Cincinnati, has been selected by the European Committee on Organization of the International Periodical Congress of Gynecology and Obstetrics, as honorary president of the meeting of that body, to be held in the city of Geneva, Switzerland, the first week in September in this year.

---

**Dr. J. G. Daniel,** Gilmer, Texas, in renewing his subscription for the twelfth consecutive year says: "I have read every number of the JOURNAL, and am unwilling to try to get along without it. May it be the power in the future against quackery and unprofessional conduct that it has ever been in the past."

---

**The** Great Texas Fruit Palace, at Tyler Texas, will be open from July 8th to 22d, inclusive; and the State Horticultural Society will hold its annual meeting in the Palace building, on the 8th, 9th and 10th. The fruit palace, it will be seen, will be open during the session of the East Texas Medical Society,—a strong additional attraction.

---

**American Medical Association.**—The President-elect of the American Medical Association is Prof. Nicholas Senn; Dr. Geo. Sternburg, Surgeon General U. S. A., was elected Vice-President. The next meeting is to be held at Philadelphia. The addresses are to be delivered by Flint, Keen and Cochrane.

We had hoped to see that noble old Roman, Jerome Cochran, of Alabama, honored by the Presidency, but—

**Our distinguished** and genial friend, Col. Francis Coles Ford, M. D., Surgeon Medical Director 1st Division Texas Volunteer Guard, a leading physician of Nacogdoches, Texas, was married on 25th May (ult.) at Beaumont, Texas, to Miss Jean Coleman Thompson, of that city.

The JOURNAL extends its hearty congratulation to the Colonel and Coloneless and wishes them a whole lot of happiness.

---

**Dr. T. J. Wagley,** of Cleburne, Tex., accompanied by his wife, sailed from New York for Europe on the 25th ult. They will spend a year in Berlin. The trip is made especially in the interest of Mrs. Wagley's health, which is much impaired, and as a former visit to Berlin was thought to be of great benefit to her, they have gone there again in the hope of improvement. The doctor will study in the great clinics and hospitals of Berlin, and will occasionally contribute a paper to the "Red-Back."

---

**Dr. Peterson's paper** will be read with pleasure and profit. We are pleased to be able to lay it before our readers, and to say that it was contributed by the author in person to the TEXAS MEDICAL JOURNAL, and will appear exclusively in that journal. The strong endorsement given by this high authority to thyroid extract in certain diseases must stimulate the demand for a reliable preparation of that article; and in this connection the JOURNAL calls attention to the fact that Parke, Davis & Co., the physicians' friend and purveyor, the pioneer in the animal extract business, has upon the market a superior prepartion made with care and attention to every detail in their laboratory, and furnished the profession under full guarantee of purity and strength. Write them; mention the "Red Back."

---

**Fifteen Cent Doctors** (two for a quarter).—Who was it who said that every man has his price? Business is business, and an enterprising firm in New York believing this, have—we suppose—compiled statistics and *averaged* the price—as regards the doctors, at least, and have fixed it at *fifteen cents!* They propose to bribe the doctors to prescribe their preparations by giving the doctor a rebate or percentage on each sale. It is a grand scheme! Worthy of a "Napoleon of Finance." It is called "the Doctors' Benevolent Fund," and the scheme is unfolded in a "personal" letter to the doctors. Every doctor in Austin has received one. The firm deprecate the fact that doctors are called upon frequently to do gratuitous work, and by this plan they propose to "divide profits with them"—to relieve the doctor of this burden they have certain preparations on the market, at fifty cents a bottle, They will furnish the doctor with a neat little prescription book, with a coupon to each prescription blank. Each coupon calls for fifteen cents. The doctor prescribes the firm's preparation; the patient goes and buys the medicine, and the doctor forwards the coupon to the firm in New York and gets it cashed. Bully!

Bnt did anybody ever hear of euch a piece of cool, deliberate effrontery? State prison is too good for such fellows.

---

**Caps and Gowns.**—The Faculty of the Medical Department of the University of Texas, by resolution, adopted the cap and gown to be worn at the annual commencement, by both Faculty and class. At the recent commencement there were forty-two graduates. We were not present on the occasion, but are informed that it was most impressive. The grave and reverend Professors wore full-flowing robes of silk, with the Oxford caps, irreverently called the "mortar-board," while the graduates were attired in less expensive, though equally expansive gowns, all with the balloon sleeve. A funny episode occurred. Two students rebelled; refused positively, as they said, to "make a guy of themselves," i. e., to don the cap and—robes. To get even with them, in calling the roll of graduates (and as each name was called, the young gents stepped forward and received their sheepskins), the dean skipped the names of the two recalcitrants; but at the close of the exercises, he stated that there were two other gentlemen who had passed examination and were entitled to the degree—calling their names—but that they preferred to not appear upon the stage with the others of the class; and their diplomas were awarded in private, so we were told.

---

Small-pox has been cropping out here and there in Texas within the recent past, having been introduced in the first place from New Orleans. It must be that there has been a relaxation of the usual vigilance of the health authorities of our sister State; small-pox ought not to get out. Instead of everybody shutting doors against an infection, the infection must be circumscribed and not allowed to escape; the order is reversed from the old days of shot gun; at least that's the way it is done in

Texas. As soon as a case appears it is quarantined, and all who have been knowingly exposed are quarantined also. In that way there are usually very few secondary cases. But it got a right good start in Smith county before it was discovered; and in all thirty-eight cases occurred. What we wished to call attention to, though, in this connection, was the remarkably small mortality. Only one death occurred, out of thirty-eight cases treated, and that was in a man of seventy-five years of age,—a case of the hemorrhagic variety. Dr. D. H. Connally, the county physician of Smith county, deserves much credit for such successful treatment. The average death rate of small-pox is about 25 per cent.; at Camp Jenner, amongst the refugee negroes, it was nearly 30 per cent; a fact attributable, no doubt, to the use of tents with two and three cases in each, and the sides pegged down. The Texas authorities do these things better; treat small-pox as nearly in the open air as possible—weather permitting.

The scare is about over, however, now, the last cases reported being in Liberty county, infection supposed to have been carried from Orange, where, we learn, the county physician employed a negro doctor to do what he ought to have done himself.

---

**In all the** Southwestern States, consisting of Texas, Louisiana, Mississippi, Arkansas, Oklahoma and the Indian Territory, the largest circulation credited to any publication devoted to medicine and surgery is accorded to the TEXAS MEDICAL JOURNAL, a monthly, published at Austin, Texas, and the publishers of the American Newspaper Directory will guarantee the accuracy of the circulation rating accorded to this paper by a reward of one hundred dollars, payable to the first person who successfully assails it.—*From Printers' Ink, issue of April 29th, 1896.*

---

**I. & G. N. Excursions.**—For the Christian Endeavor meeting, San Antonio, June 9–11; State Teachers' Association meeting, Austin, June 16–19, and B. Y. P. U., and State Sunday-School Convention, San Antonio, June 23–27, the I. & G. N. will make reduced rates to $5.00 maximum for round trip.

Excursions to Monterey and City of Mexico via Laredo will be run following each event.

Call on agent for full information.

D. J. PRICE, A. G. P. A.,
Palestine, Texas.

**Political Conventions.**—For the following political conventions, the Santa Fe will make round-trip rates of one fare from all of its Texas and Indian Territory points:

National Republican Convention, St. Louis, Mo., June 16th, 1896.

National People's Convention, St. Louis, Mo., July 22nd, 1896.

For particulars as to limits and time cards, call upon any Santa Fe agent or write to

W. S. KEENAN,
General Passenger Agent, Galveston.

---

**CIRCULAR TO PHYSICIANS.**

---

**Eye and Ear Charity Hospital.**

---

Through the co-operation of some of the good women of Texas, there has been founded in the city of Austin an Eye and Ear Charity Hospital for patients who are unable to pay for board and treatment. Arrangements have also been made to receive and treat patients who are able to pay. Only trained nurses are employed, and the surgeon in charge is daily assisted by Dr. Jos. S. Wooten.

I take this method of announcing to the profession that the hospital was opened September 1st, 1895, and has been in operation since that date. Your co-operation is respectfully solicited, and a cordial invitation is extended to you, when in the city, to visit the hospital and see for yourself that it is properly conducted.

For further information address

H. L. HILGARTNER, M. D.,
Surgeon in Charge.

